# Visfatin induces MUC8 and MUC5B expression via p38 MAPK/ROS/NF-κB in human airway epithelial cells

**DOI:** 10.1186/1423-0127-21-49

**Published:** 2014-05-20

**Authors:** Si-Youn Song, Eun Chae Jung, Chang Hoon Bae, Yoon Seok Choi, Yong-Dae Kim

**Affiliations:** 1 Department of Otorhinolaryngology-Head and Neck Surgery, College of Medicine, Yeungnam University, Daegu, Republic of Korea; 2 Regional Center for Respiratory Diseases, Yeungnam University Medical Center, Daegu, Republic of Korea

**Keywords:** Visfatin, p38 MAPK, ROS, NF-κB, MUC8, MUC5B, Epithelial cell

## Abstract

**Background:**

Among a variety of inflammatory mediators, visfatin is a proinflammatory adipocytokine associated with inflammatory reactions in obesity, metabolic syndrome, chronic inflammatory disease, and autoimmune disease. However, the biological role of visfatin in secretion of major mucins in human airway epithelial cells has not been reported. Therefore, this study was conducted in order to investigate the effect and the brief signaling pathway of visfatin on MUC8 and MUC5B expression in human airway epithelial cells.

**Results:**

Visfatin significantly induced MUC8 and MUC5B expression. Visfatin significantly activated phosphorylation of p38 MAPK. Treatment with SB203580 (p38 MAPK inhibitor) and knockdown of p38 MAPK by siRNA significantly blocked visfatin-induced MUC8 and MUC5B expression.Visfatin significantly increased ROS formation. Treatment with SB203580 significantly attenuated visfatin-induced ROS formation. Treatment with NAC (ROS scavenger) and DPI (NADPH oxidase inhibitor) significantly attenuated visfatin-induced MUC8 and MUC5B expression. However, treatment with NAC and DPI did not attenuate visfatin-activated phosphorylation of p38 MAPK. Visfatin significantly activated the phosphorylation of NF-κB. Treatment with PDTC (NF-κB inhibitor) significantly attenuated visfatin-induced MUC8 and MUC5B expression.

**Conclusions:**

These results suggest that visfatin induces MUC8 and MUC5B expression through p38 MAPK/ROS/NF-κB signaling pathway in human airway epithelial cells.

## Background

Mucins, highly glycosylated proteins, are the major components of mucus. Among the mucins, MUC8, MUC5B, and MUC5AC are representative secretory mucin genes in the human airway [1,2]. MUC8, MUC5B, and MUC5AC expression is increased by a variety of inflammatory mediators in airway inflammatory diseases such as chronic bronchitis [3].

Among a variety of inflammatory mediators, visfatin is a proinflammatory adipocytokine that is preferentially produced by visceral adipose tissue: visfatin is highly enriched in visceral fat and its expression level in plasma increases during development of obesity [4,5]. Although the biological role of visfatin is not entirely clear, visfatin is known to have insulin-mimetic action because it reduces blood glucose level [6]. Several recent studies have reported an association of visfatin with inflammation and oxidative stress response: expression of visfatin is upregulated during activation of immune cells such as monocytes, macrophages, dendritic cells, T cells, and B cells, as well as in amniotic epithelial cells upon stimulation with lipopolysaccharide, TNF-α, IL-1β, or IL-6 [7-10]. And visfatin increases expression of inflammatory adhesion molecules in vascular endothelial cells in ROS-dependent manner through NF-κB signaling pathway [5]. Therefore, it could be hypothesized that visfatin may play a role in expression of mucin gene in human airway epithelial cells. However, the biological role of visfatin in secretion of major mucins in human airway epithelial cells has not been reported. For the this reason, this study was conducted in order to investigate the effect and the brief signaling pathway of visfatin on MUC8 and MUC5B expression in human airway epithelial cells.

## Methods

### Materials

Visfatin was purchased from Biovision Inc. (Mountain View, CA, USA). RT-PCR kits were obtained from Applied Biosystems (Foster City, CA, USA). Real-time PCR kits were obtained from Roche Applied Science (Mannheim, Germany). Enhanced chemiluminescence kit was obtained from Perkin Elmer Life Sciences (Boston, MA, USA). ERK1/2, phospho-ERK1/2, p38, phospho-p38, NF-κB, and phosphor-NF-κB were purchased from Cell Signaling Technology (Danvers, MA, USA). The specific inhibitor, U0126 was purchased from Calbiochem (San Diego, CA, USA), SB203580 was purchased from BIOMOL (Plymouth Meeting, PA, USA), and NAC, DPI, and PDTC were purchased from Sigma-Aldrich (St. Louis, MO, USA). Predesigned siRNA targeting p38 MAPK, negative control siRNA for p38 MAPK, OPTI-MEM I Reduced Serum Medium, and Lipofectamine 2000 were purchased from Invitrogen Corporation (Carlsbad, CA, USA).

For the primary culture, nasal mucosa was obtained from normal inferior turbinate samples from 10 patients undergoing augmentation rhinoplasty who had no personal or family history of allergy, and who had negative results on skin-prick tests to 20 common airborne allergens and on multiple simultaneous allergen tests. This study was approved by the institutional review board for human studies at the Yeungnam University Medical Center and written informed consent was obtained from each patient.

### Cell culture and treatment

Human NCI-H292 airway epithelial cells (American Type Culture Collection, Manassas, VA, USA) were cultured in RPMI 1640 medium (Invitrogen Corporation) supplemented with 2 mM L-glutamine, 100 U/mL penicillin, 100 μg/mL streptomycin, and 10% fetal bovine serum (Hyclone Laboratories, Logan, UT, USA). The cells were grown at 37˚C in 5% CO_2_ fully humidified air and subcultured twice weekly. When the cultures had reached confluence, the cells were incubated in RPMI 1640 medium containing 0.5% fetal bovine serum for 24 h. The cells were then rinsed with serum-free RPMI 1640 medium and exposed to the indicated concentrations of visfatin.

For the primary culture of human nasal epithelial cells, the nasal mucosal tissue was washed with PBS and immersed in dispase (Boehringer Mannheim Biochemica, Mannheim, Germany) for 90 min. The tissue was scraped off the surface of the nasal mucosa using a scalpel; it was then added to 1% PBS and filtered through a mesh. The cells were seeded in a 24-well plate at 2.5 × 10^5^ cells/well, followed by incubation with EpiLife medium (Cascade Biologics, Portland, OR, USA) and human keratinocyte growth supplement (5 mL/500 mL of medium, Cascade Biologics). When the cultures had reached confluence, the cells were exposed to the indicated concentrations of visfatin. To investigate the brief signaling pathway of mucin gene expression, human NCI-H292 airway epithelial cells and human nasal epithelial cells were pretreated with U0126, SB203580, PDTC, NAC, or DPI for 1 h before exposure to visfatin. For the controls, human NCI-H292 airway epithelial cells and human nasal epithelial cells were incubated with the medium alone for the same amount of time.

### RT-PCR and real time PCR analysis of MUC16, MUC8, MUC5B, MUC5AC, and MUC4 mRNA expression

Total RNA was isolated from the cultured cells according to the manufacturer’s instructions (Applied Biosystems). Each sample was reverse transcribed into cDNA using the GeneAmp RNA PCR Core Kit (Applied Biosystems). The primer sequences and conditions used were according to previously published protocols for MUC16, MUC8, MUC5B, MUC5AC, and MUC4 [11,12]. The PCR products were electrophoresed on a 2% agarose gel, stained with ethidium bromide, and visualized by UV fluorescence. Semiquantitative analysis of the RT-PCR product was performed on the scanned gel images, and the intensity of the PCR product was measured using commercially available imaging software (Scion, Frederick, MD, USA). The relative intensity of the individual bands on the gel image was determined as the ratio of the intensities of each MUC16, MUC8, MUC5B, MUC5AC, and MUC4 to the intensity of GAPDH.

Real-time PCR was performed using the LC Fast Start DNA Master SYBR Green kit (Roche Applied Science) using 0.5 μL of cDNA, corresponding to 25 ng of total RNA in a 10 μL final volume, 2.5 mM MgCl_2_ and 0.5 μM of each primer (final concentration). Quantitative PCR was performed using a LightCycler (Roche Applied Science) for 45 cycles at 95˚C for 10 s, specific annealing temperature for 5 s and 72˚C for 10 s. Data were normalized to GAPDH. Amplification specificity was evaluated using a melting curve, following the manufacturer’s instructions (Roche Applied Science).

### ELISA analysis of MUC8 and MUC5B proteins

MUC8 and MUC5B protein levels were determined by ELISA. Samples of supernatant or cell lysates from human NCI-H292 airway epithelial cells were prepared in PBS at several dilutions, and each sample was incubated at 40˚C in a 96-well plate until dry. The plates were then washed three times with PBS, blocked with 2% bovine serum albumin for 1 h at room temperature, washed again three times with PBS, and incubated with primary antibody for MUC8 or MUC5B (Santa Cruz Biotechnology, Santa Cruz, CA, USA), followed by dilution at 1:200 with PBS containing 0.05% Tween 20 for 1 h. The wells were then washed three times with PBS, horseradish peroxidase-conjugated secondary antibody for MUC8 or MUC5B (Santa Cruz Biotechnology) was dispensed into each well. After 4 h, color was developed using 3,3’, 5,5’-tetramethylbenzidine peroxidase solution and stopped with 2 N-H_2_SO_4_. Optical density was measured at 450 nm using an ELISA reader (EL800; BIO-TEK Instruments, Winooski, VT, USA).

### Western blot analysis of p38 MAPK, ERK1/2 MAPK, and NF-κB

Samples of cells from human NCI-H292 airway epithelial cells were seeded in wells of a 6-well plate and treated with visfatin. The cells were exposed to trypsin and formed into pellets at 700 g at 4˚C pellets were resuspended in lysis buffer. The preparation was then clarified by centrifugation, and the supernatant was saved as a whole-cell lysate. Proteins (20 μg) were separated using 10% reducing sodium dodecyl sulfate-polyacrylamide gel electrophoresis and electroblotted onto a nitrocellulose membrane. The membrane was blocked with 5% nonfat dry milk, followed by incubation with the indicated primary antibody for p38, ERK1/2, or NF-κB (Cell Signaling Technology) for 4 h. Subsequently, the membrane was incubated for 1 h with secondary antibody for p38, ERK1/2, or NF-κB (Cell Signaling Technology) conjugated to horseradish peroxidase, and developed using an enhanced chemiluminescence kit. Bands were detected after exposure to x-ray film for 10 s.

### Cell transfection with siRNA for p38 MAPK

Sequences of each siRNA were as follows: p38 MAPK; forward: AUG AAU GAU GGA CUG AAA UGG UCU G and reverse: CAG ACC AUU UCA GUC CAU CAU UCA U. The transfection rate of p38 MAPK siRNA was verified to be over 90% in human NCI-H292 airway epithelial cells. Transfection was performed according to the manufacturer’s protocol (Invitrogen Corporation). Briefly, the cells were seeded in wells of a 6-well plate at 1 × 10^5^ cells/well and incubated in RPMI 1640 medium. When the cells had reached confluence, OPTI-MEM I Reduced Serum Medium was added. Then, p38 MAPK siRNA and Lipofectamine 2000 were incubated together in OPTI-MEM I Reduced Serum Medium to form a p38 MAPK siRNA-Lipofectamine complex. The p38 MAPK siRNA-Lipofectamine complex-containing medium was added to each well containing the cells to a final p38 MAPK siRNA concentration of 20 nM. After 24 h of transfection with p38 MAPK siRNA, the cells were exposed to the indicated concentrations of visfatin and then harvested for RT-PCR analysis of MUC8 and MUC5B mRNA expression. The same procedure was performed with negative control siRNA: the cells were transfected with Lipofectamine 2000 only in negative control siRNA.

### Flow cytometric measurement of ROS

Human NCI-H292 airway epithelial cells were loaded with 50 μM 2,7-DCF-DA (Sigma-Aldrich) for 40 min, and were then washed three times with PBS before they were harvested. The washed cells were resuspended in 1 mL of PBS and kept on ice until flow cytometric analysis was started. Measurement of ROS formation was performed immediately by flow cytometry using FACSort (BD bioscience, Rutherford, NJ, USA) with a 488-nm excitation beam. The signals were obtained using a 530-nm band-pass filter for 2,7-DCF-DA. Each determination was based on the mean fluorescence intensity of 5,000 cells.

### Statistical analysis

Commercially available software (SPSS, version 10.0; SPSS Inc, Chicago, IL, USA) was used in performance of statistical analysis. The mean for each of the obtained quantitative values was calculated. Comparisons were made using the Student’s *t*-test. For all tests, a *p* value of less than 0.05 was considered statistically significant.

## Results

### Visfatin induced MUC8 and MUC5B expression in NCI-H292 cells

**Figure 1:**
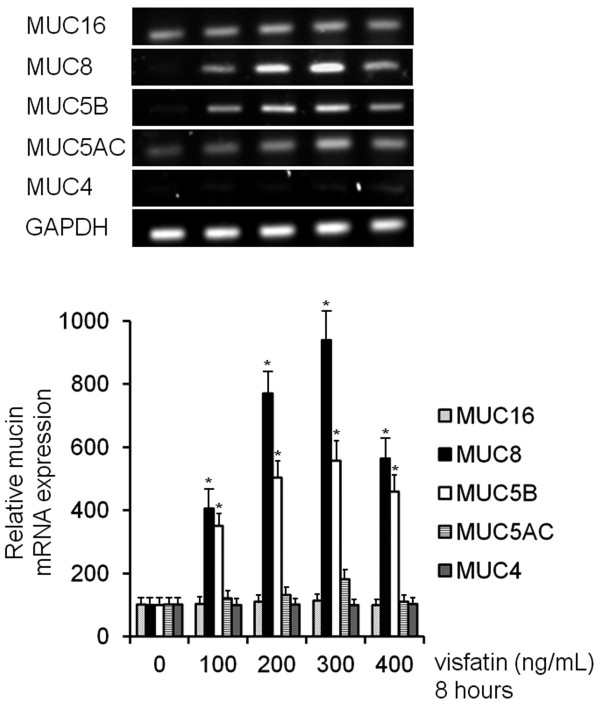
**The effects of visfatin on MUC16, MUC8, MUC5B, MUC5AC, and MUC4 expression in NCI-H292 cells.** RT-PCR showed that visfatin induced remarkable MUC8 and MUC5B mRNA expression. However, visfatin did not definitely induce MUC16, MUC5AC, and MUC4 mRNA expression. Images are representative of three separate experiments performed in triplicate. Bars indicate the mean ± S.D. of three independent experiments performed in triplicate. *P < 0.05 compared with zero value.

**Figure 2:**
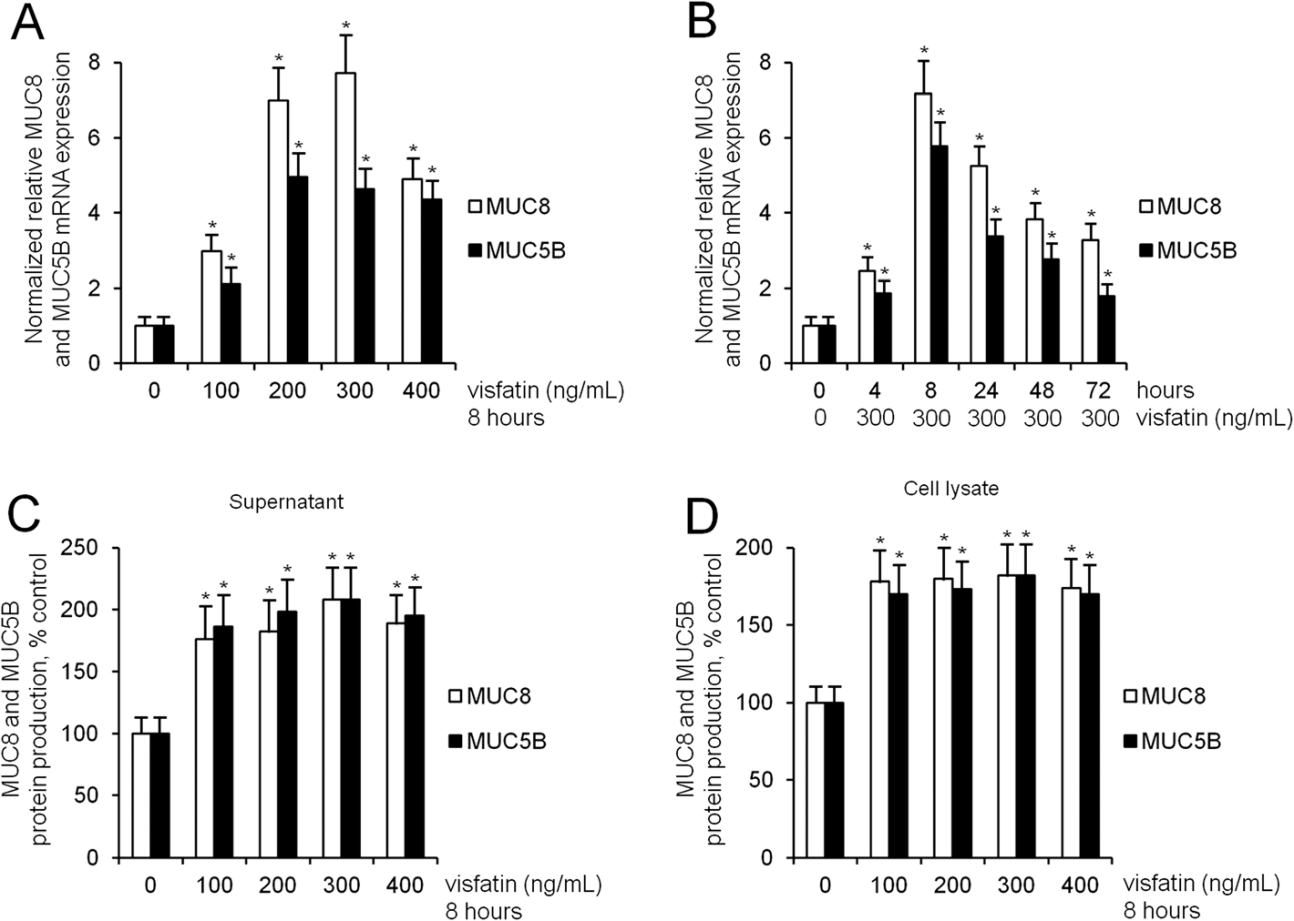
**The effects of visfatin on MUC8 MUC5B expression in NCI-H292 cells. (A-D)** Real time RT-PCR and ELISA showed that visfatin significantly increased MUC8 and MUC5B expression: visfatin-induced MUC8 expression was significantly stronger than visfatin-induced MUC5B expression, and MUC8 and MUC5B mRNA expression peaked at 8 h after treatment with visfatin (300 ng/mL). Images are representative of three separate experiments performed in triplicate. Bars indicate the mean ± S.D. of three independent experiments performed in triplicate. *P < 0.05 compared with zero value.

To investigate the effect of visfatin on MUC16, MUC8, MUC5B, MUC5AC, and MUC4 expression in human NCI-H292 airway epithelial cells, the cells were incubated with different doses of visfatin for 8 h. Result of RT-PCR showed remarkable visfatin-induced MUC8 and MUC5B mRNA expression: visfatin-induced MUC8 mRNA expression was significantly stronger than visfatin-induced MUC5B mRNA expression. However, visfatin did not definitely induce MUC16, MUC5AC, and MUC4 mRNA expression (Figure 1, *P* < 0.05). Real time RT-PCR and ELISA analysis were performed to investigate the effect of visfatin on MUC8 and MUC5B expression in a dose- and time-dependent manner. The cells were treated with different doses of visfatin for 8 h or visfatin (300 ng/mL) for variable times. The results also showed a significant increase in MUC8 and MUC5B mRNA expression and protein production by treatment with all dosages of visfatin: visfatin-induced MUC8 expression was significantly stronger than visfatin-induced MUC5B expression, and MUC8 and MUC5B mRNA expression peaked at 8 h after treatment with visfatin (300 ng/mL) (Figure 2A-D, *P* < 0.05).

### p38 MAPK was involved in visfatin-induced MUC8 and MUC5B expression in NCI-H292 cells

**Figure 3:**
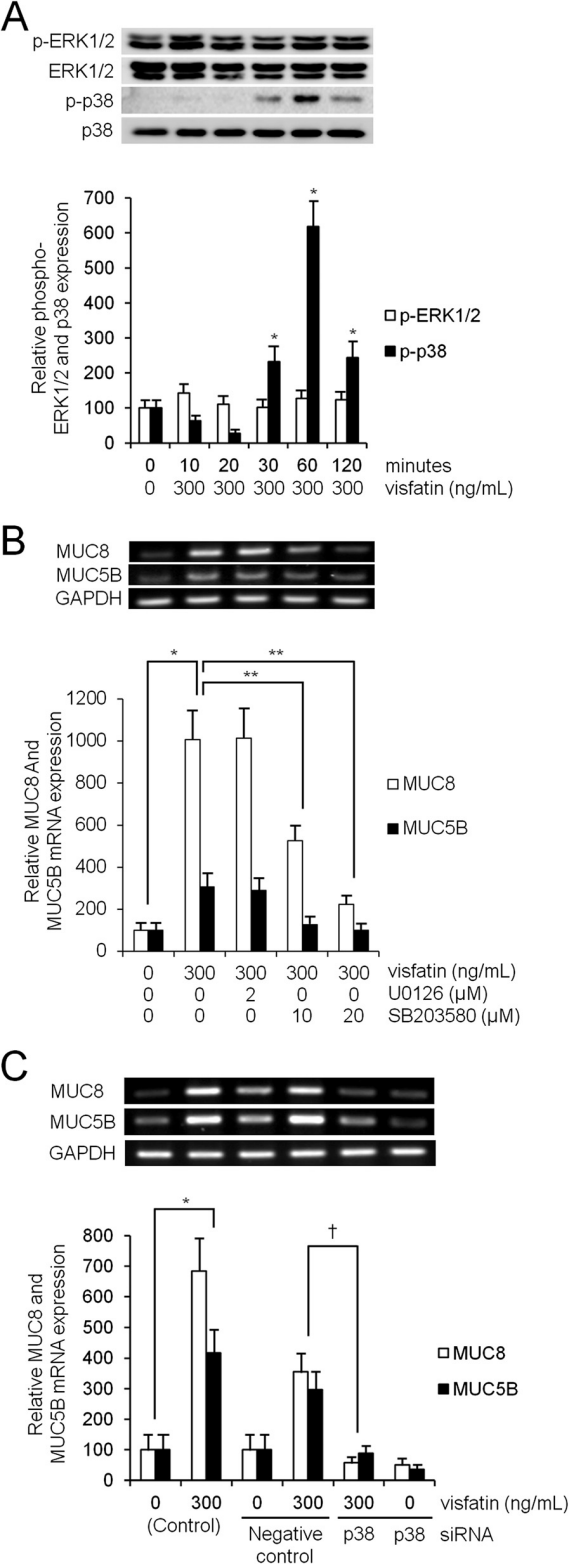
**The phosphorylation of p38 MAPK in visfatin-induced MUC8 and MUC5B expression in NCI-H292 cells. (A)** Western blot analysis showed that visfatin activated phosphorylation of p38 MAPK; however, visfatin did not activate phosphorylation of ERK1/2 MAPK. **(B)** RT-PCR showed that SB203580 inhibited visfatin-induced MUC8 and MUC5B expression; however, U0126 did not inhibit visfatin-induced MUC8 and MUC5B expression. **(C)** RT-PCR showed the knockdown of p38 MAPK by siRNA significantly blocked visfatin-induced MUC8 and MUC5B mRNA expression. Images are representative of three separate experiments performed in triplicate. Bars indicate the mean ± S.D. of three independent experiments performed in triplicate. *P < 0.05 compared with zero value. **P < 0.05 compared with visfatin alone, ^†^*P* < 0.05 compared with negative control.

To evaluate the brief intracellular mechanisms of visfatin-induced MUC8 and MUC5B expression, ERK1/2, or p38 MAPK signaling pathways were investigated in order to determine whether they were capable of activating MUC8 and MUC5B expression in human NCI-H292 airway epithelial cells. Results of Western blot analysis showed that visfatin activated phosphorylation of p38 MAPK; however, visfatin did not activate phosphorylation of ERK1/2 MAPK (Figure 3A, *P* < 0.05). To confirm ERK1/2, or p38 MAPK signaling pathways in visfatin-induced MUC8 and MUC5B expression, the cells were treated with U0126 as a specific ERK1/2 inhibitor or SB203580 as a p38 MAPK inhibitor for 1 h before exposure to visfatin for 8 h. Results of RT-PCR showed that SB203580 inhibited visfatin-induced MUC8 and MUC5B expression; however, U0126 did not inhibit visfatin-induced MUC8 and MUC5B expression (Figure 3B, *P* < 0.05). In addition, cell transfection with siRNA was performed in order to confirm whether phosphorylation of p38 MAPK was associated with visfatin-induced MUC8 and MUC5B mRNA expression. Results of RT-PCR showed that the knockdown of p38 MAPK by siRNA significantly blocked visfatin-induced MUC8 and MUC5B mRNA expression (Figure 3C, *P* < 0.05).

### ROS was involved in visfatin-induced MUC8 and MUC5B expression in NCI-H292 cells

**Figure 4:**
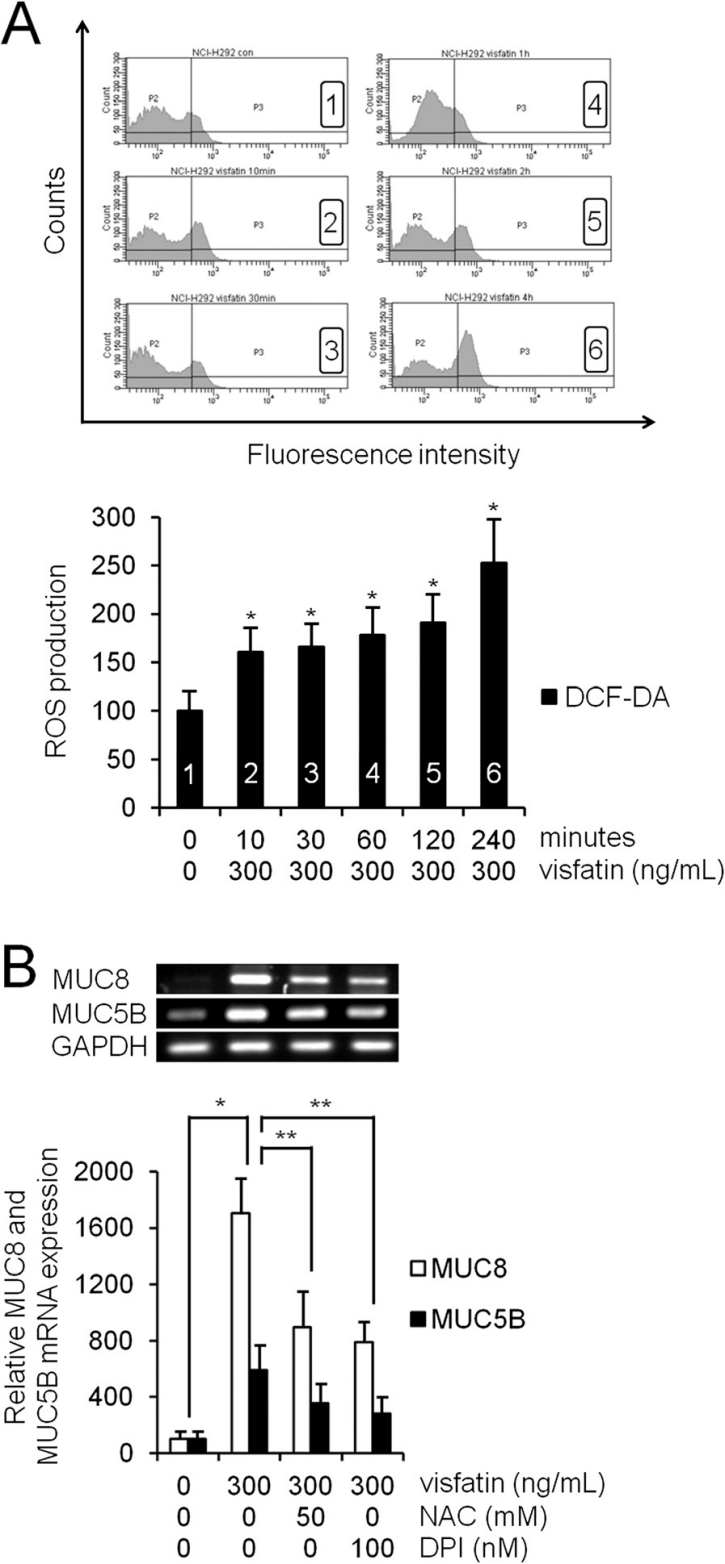
**The role of ROS in visfatin-induced MUC8 and MUC5B expression in NCI-H292 cells. (A)** Flow cytometry showed that visfatin (300 ng/mL) significantly induced ROS formation, especially after 2 h. **(B)** RT-PCR showed that both NAC and DPI significantly attenuated visfatin-induced MUC8 and MUC5B mRNA expression. Images are representative of three separate experiments performed in triplicate. Bars indicate the mean ± S.D. of three independent experiments performed in triplicate. *P < 0.05 compared with zero value. **P < 0.05 compared with visfatin alone.

**Figure 5:**
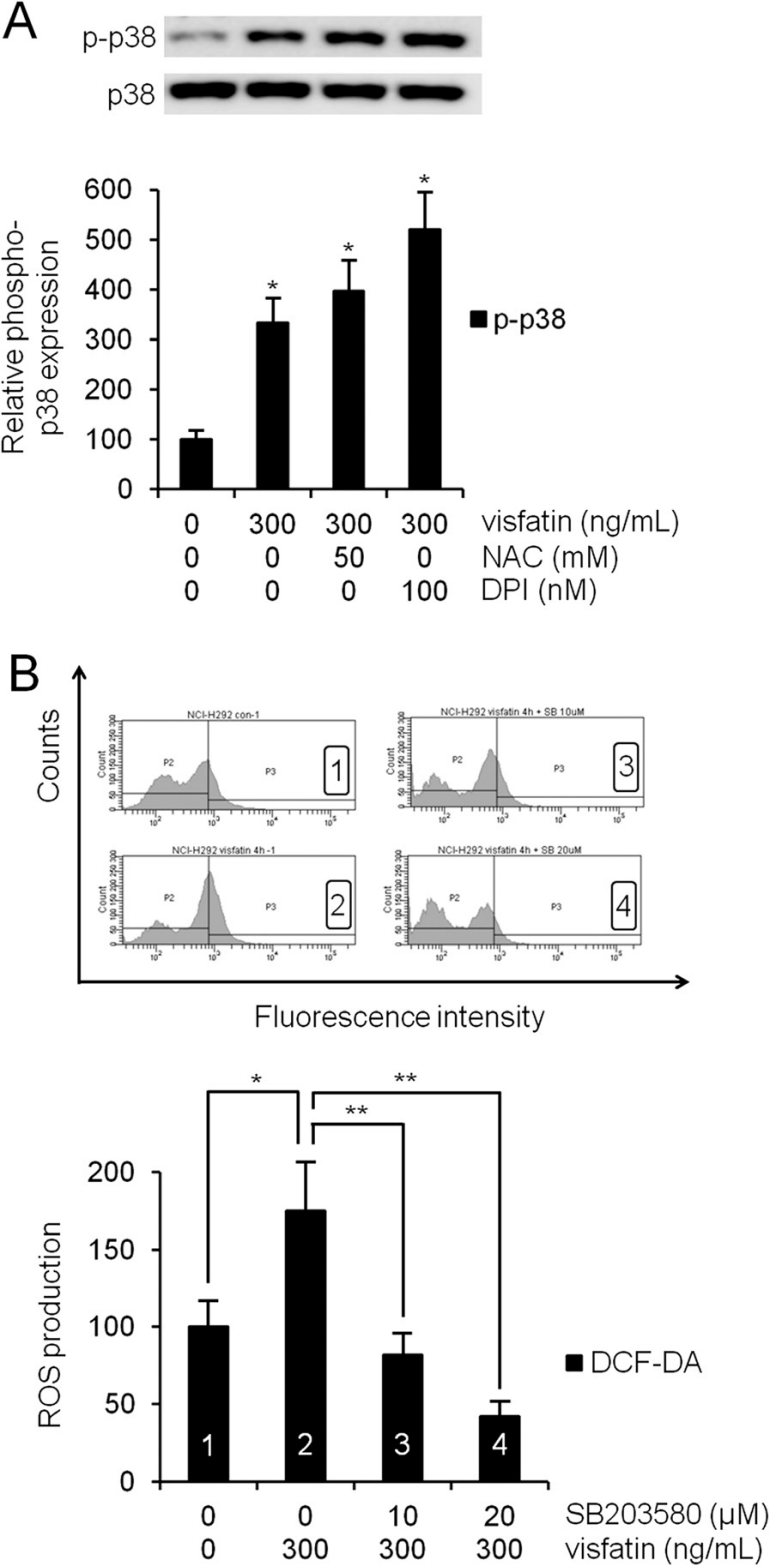
**The role of ROS in visfatin-activated phosphorylation of p38 MAPK in NCI-H292 cells. (A)** Western blot showed that both NAC and DPI did not attenuate visfatin-activated phosphorylation of p38 MAPK. **(B)** Flow cytometry showed that SB203580 significantly inhibited visfatin-induced ROS formation. Images are representative of three separate experiments performed in triplicate. Bars indicate the mean ± S.D. of three independent experiments performed in triplicate. *P < 0.05 compared with zero value. **P < 0.05 compared with visfatin alone.

To examine visfatin-induced ROS formation, human NCI-H292 airway epithelial cells were preincubated with redox-sensitive fluorescent dye 2,7-DCF-DA. Compared with control, flow cytometry showed that visfatin (300 ng/mL) significantly induced ROS formation, especially after 2 h (Figure 4A, *P* < 0.05). To investigate the role of ROS in visfatin-activated phosphorylation of p38 MAPK in MUC8 and MUC5B expression, the cells were pretreated with NAC as a ROS scavenger, or DPI as an NADPH oxidase, for 1 h before exposure to visfatin. Results of RT-PCR showed that both NAC and DPI significantly attenuated visfatin-induced MUC8 and MUC5B mRNA expression (Figure 4B, *P* < 0.05). However, results of Western blot showed that neither NAC nor DPI attenuated visfatin-activated phosphorylation of p38 MAPK (Figure 5A, *P* < 0.05). To confirm the correlation between p38 MAPK and ROS, the cells were pretreated with SB203580, as a specific inhibitor of p38 MAPK, 1 h before exposure to visfatin. Flow cytometry showed that SB203580 significantly attenuated visfatin-induced ROS formation (Figure 5B, *P* < 0.05).

### NF-κB was involved in visfatin-induced MUC8 and MUC5B expression in NCI-H292 cell*s*

**Figure 6:**
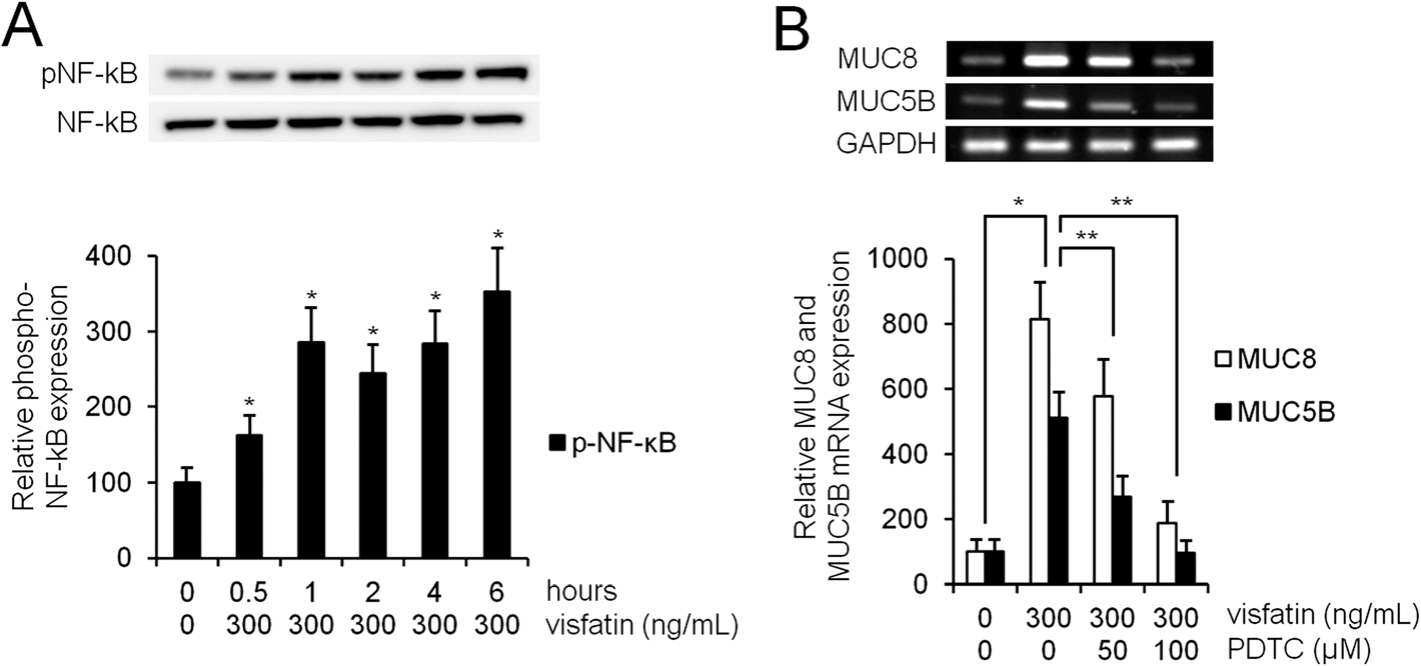
**The role of NF-κB in visfatin-induced MUC8 and MUC5B expression in NCI-H292 cells. (A)** Western blot analysis showed that visfatin significantly activated the phosphorylation of NF-κB. **(B)** RT-PCR showed that PDTC significantly attenuated visfatin-induced MUC8 and MUC5B mRNA expression. Images are representative of three separate experiments performed in triplicate. Bars indicate the mean ± S.D. of three independent experiments performed in triplicate. *P < 0.05 compared with zero value. **P < 0.05 compared with visfatin alone.

Western blot analysis was performed to investigate the effect of visfatin on NF-κB. Human NCI-H292 airway epithelial cells were treated with visfatin. The results showed that visfatin significantly activated the phosphorylation of NF-κB (Figure 6A, *P* < 0.05). To investigate the role of NF-κB in visfatin-induced MUC8 and MUC5B expression, the cells were pretreated with PDTC as an inhibitor of NF-κB for 1 h before exposure to visfatin. Results of RT-PCR showed that PDTC significantly attenuated visfatin-induced MUC8 and MUC5B mRNA expression (Figure 6B, *P* < 0.05).

### Visfatin induced MUC8 and MUC5B expression via p38 MAPK in primary cultures of normal nasal epithelial cells

**Figure 7:**
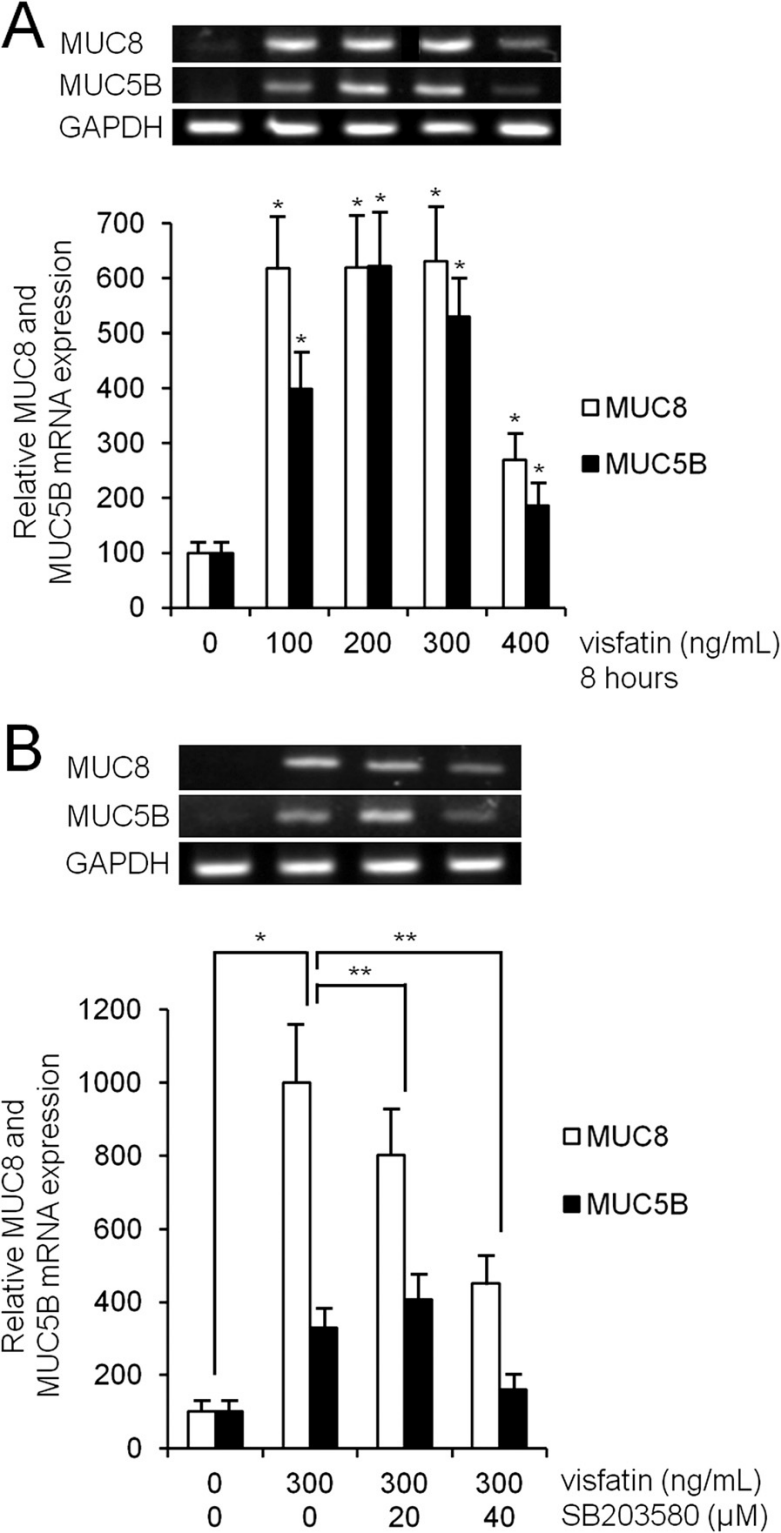
**The effect of visfatin on MUC8 and MUC5B expression in normal nasal epithelial cells. (A)** RT-PCR showed that a significant increase of MUC8 and MUC5B mRNA expression by treatment with all dosages of visfatin. **(B)** RT-PCR showed that visfatin-induced MUC8 and MUC5B mRNA expression were significantly attenuated by pretreatment with SB203580 as a p38 MAPK inhibitor. Images are representative of three separate experiments performed in triplicate. Bars indicate the mean ± S.D. of three independent experiments performed in triplicate. *P < 0.05 compared with zero value. **P < 0.05 compared with visfatin alone.

To investigate the effect of visfatin on MUC8 and MUC5B expression in primary cultures of normal nasal epithelial cells, the cells were incubated with different doses of visfatin for 8 h. Results of RT-PCR showed a significant increase in MUC8 and MUC5B mRNA expression by treatment with all dosages of visfatin (Figure 7A, *P* < 0.05), and SB203580 as a p38 MAPK inhibitor significantly attenuated visfatin-induced MUC8 and MUC5B mRNA expression (Figure 7B, *P* < 0.05).

## Discussion

Visfatin, a 52-kDa protein, was previously identified as a PBEF. PBEF is secreted by human peripheral blood lymphocytes, and acts like a nicotinamide phosphoribosyl transferase, which is involved in nicotinamide adenine dinucleotide biosynthesis and is related to glucose and lipid metabolism in humans [13,14]. PBEF was recently found in high levels in visceral fat, and was renamed visfatin. The biological roles of visfatin include an insulin mimetic effect, cytokine and immunomodulator, enzyme, and anti-apoptotic effect. As an immunomodulator, visfatin induces dose-dependent up-regulation of the pro- and anti-inflammatory cytokines, IL-1β, IL-6, IL-10, and TNF-α in human monocytes: visfatin is associated with sepsis, acute lung disease, atherosclerosis, and cancer [13,15]. However, the biological role of visfatin in secretion of major mucins in human airway epithelial cells has not been reported. Therefore, it could be hypothesized that visfatin may play a role in mucin gene expression in human airway epithelial cells. This study was conducted in order to determine whether visfatin might regulate expression of the major secretory airway mucin genes in airway epithelial cells. The results of this study showed that visfatin significantly increased MUC8 and MUC5B expression. However, visfatin did not definitely induce MUC16, MUC5AC, and MUC4 mRNA expression. These results suggest that visfatin has up-regulation of MUC8 and MUC5B expression, like an inflammatory mediator: lipopolysaccharide, TNF α, and IL-1β.

Within the signaling pathway, visfatin induces vascular endothelial growth factor, and production of matrix metalloproteinases via MAPK [8,16]. In addition, MUC8 or MUC5B expression is induced in response to a wide variety of stimuli, including nerve activation and inflammatory cytokines, such as IL-1β, IL-6, TNF-α, and prostaglandin E_2_ through a process involving p38 or ERK1/2 MAPK activation [11,17,18]. And insulin-like growth factor-1, which has an insulin mimetic effect like visfatin, induces MUC8 and MUC5B expression via ERK1 and p38 MAPK signaling pathway in human airway epithelial cells [19]. Therefore, this study focused on visfatin-induced MUC8 and MUC5B expression via the p38 or ERK1/2 MAPK signaling pathway. The results of this study showed that visfatin activated phosphorylation of p38 MAPK. SB203580 inhibited visfatin-induced MUC8 and MUC5B expression. In addition, the knockdown of p38 MAPK by siRNA significantly blocked visfatin-induced MUC8 and MUC5B mRNA expression. These results suggest that visfatin induces MUC8 and MUC5B expression through the p38 MAPK signaling pathway in human airway epithelial cells.

ROS produced by cytokines, growth factors, and vasoactive agents contribute to the intracellular signaling cascades associated with inflammatory responses. ROS induce NF-κB activation by modifying the activity of one or more of the kinase enzymes in the NF-κB activation cascades [5]. Recent studies have reported that visfatin increases expression of inflammatory adhesion molecules through an ROS-dependent NF-κB signaling pathway in vascular endothelial cells [5,7,9,16,20]. Therefore, this study focused on correlation between visfatin-induced MUC8 and MUC5B expression and ROS formation via the NF-κB signaling pathway in human airway epithelial cells. The results of this study showed that visfatin significantly induced ROS formation. Treatment with SB203580 significantly attenuated visfatin-induced ROS formation. Treatment with NAC and DPI significantly attenuated visfatin-induced MUC8 and MUC5B expression. However, neither NAC nor DPI attenuated visfatin-activated phosphorylation of p38 MAPK. Visfatin significantly activated the phosphorylation of NF-κB. PDTC significantly attenuated visfatin-induced MUC8 and MUC5B expression. These results suggest that visfatin induces MUC8 and MUC5B expression through the p38 MAPK/ROS/NF-κB signaling pathway in human airway epithelial cells.

## Conclusions

In summary, the results of this study demonstrate for the first time that visfatin induces MUC8 and MUC5B expression in human airway epithelial cells. In addition, visfatin-induced MUC8 and MUC5B expression may be regulated through the p38 MAPK/ROS/NF-κB signaling pathway in human airway epithelial cells. These results provide important information demonstrating that modulation of visfatin may be an appropriate pharmacological target for control of mucus-hypersecretion in treatment of airway inflammatory diseases in patients with obesity.

## Abbreviations

DCF-DA: Dichlorodihydrofluorescein diacetate; DPI: Diphenyleneiodonium; ELISA: Enzyme-linked immunosorbent assay GAPDH, Glyceraldehyde-3-phosphate dehydrogenase; IL: Interleukin; MAPK: Mitogen-activated protein kinase; NAC: N-acetyl-cystein; NADPH: Nicotinamide adenine dinucleotide phosphate; NF-κB: Nuclear factor kappa-light-chain-enhancer of activated B cells; PBEF: Pre-B cell colony-enhancing factor; PBS: Phosphate-buffered saline; PCR: Polymerase chain reaction; PDTC: Pyrrolidine dithiocarbamate; ROS: Reactive oxygen species; RT: Reverse transcriptase; SiRNA: small interfering RNA; TNF: Tumor necrosis factor.

## Competing interests

The authors declare that they have no competing interests.

## Authors’ contributions

S-YS conceived and designed this study; ECJ drafted the manuscript and acquired data; Y-DK supervised this study and provided the critical revision of the manuscript for important intellectual content; YSC and BCH analyzed and interpreted data; S-YS and ECJ provided the administrative, technical, or material support. All authors read and approved the final manuscript.
